# Influence of Youth Baseball Players’ Pitches on Range of Motion and Ball Speed

**Published:** 2017-09

**Authors:** Seung-Won YANG, Se-Jeong KWON, Joon-Sung PARK, Tae-Young KIM, Nam-Il AN, Young-Sub LEE

**Affiliations:** 1. College of Education, Hankuk University of Foreign Studies, Seoul, Korea; 2. College of Sport Sciences, Chung-Ang University, Ansung, Korea; 3. Division of Wellbeing Physical Education, Silla University, Pusan, Korea; 4. Division of Culture Creativity, Korea University, Sejong, Korea; 5. Asia Contents Institute, Konkuk University, Seoul, Korea

## Dear Editor-in-Chief

Many studies are underway on youth baseball players shoulder ROM (range of motion) and ball speed in order to prevent them from having injuries to their shoulder joints, to work out the appropriate number of pitches, and to develop effective training programs.

After a pitch, the internal rotation of the shoulder joint (shoulder IR) will become narrow remarkably, whereas the external rotation of the shoulder joint (shoulder ER) will be extended conspicuously. This may make the rotator cuff, static structures, and periscapular muscles out of balance (1). Synchronization and coordination among upper body muscles, arm, and fingertip are crucial to have the most effective pitching motion and the fastest ball (2). Arm acceleration is from when the shoulder joint externally rotates to the full to when the ball leaves the hand. It can be done within 0.03 to 0.04 sec. When the shoulder joint reaches the full external rotation, the elbow joint begins to be extended. Its angular velocity can reach up to 2100° to 2400°/sec halfway through extension. The shoulder joint makes an external rotation at a speed of at most 7000° to 8000°/sec. It is bent toward the front of the body at an angular velocity of at most 300° to 450°/sec (3).

Regarding orthodox pitchers who pitch balls by raising their arms over their heads, namely, overhead pitchers, a 142-gram ball can max out at speed of 90 mph within 0.05 sec, flying 18.44 meters. It is equivalent to an angular velocity of about 6100°/sec. In that case, compressive load reaches about 860 N (4). This suggests that there is a need to develop shoulder exercise programs and have physical trainers at least during the season. This study aimed to compare shoulder ROM and ball speed measured before a youth baseball season and after the season to provide data necessary to develop a program intended for youth baseball players to prevent shoulder injuries and relax their shoulder joints.

This study was conducted prior to and after the youth baseball season in 2013 on 26 youth baseball players (mean 14.5 yr, 14 pitchers and 12 fielders) listed on KBA.

The ROM of the dominant shoulder joint (DSROM) was measured through the protractor pivot adjusted to the center of the shoulder joint for every subject in a supine position. Additionally, the pivot arm was adjusted and fastened onto the upper arm along with adduction and abduction. Shoulder joints of all subjects were abducted and adducted at 90° in a supine position and then their forearms were pronated. The pivot was then adjusted to the center of the elbow joint, through which the range of IR and ER were measured twice by one researcher (5). All variables are presented as mean and standard deviation (SPSS version 18.0 for Windows). Also, paired *t*-test was used to compare ball speed before and after the season. Statistical significance was defined at *P*<0.05.

The protocol was approved by the Bioethics Institutional Review Board of Silla University (Project No. 1041449-201502-HR-001).

A change in DS-ROM of youth baseball pitchers at pre-season and post-season are summarized in Table 1. The IR was measured at 31.65° before the season. It was increased to 34.03° after the season. The increase was statistically significant (*P*<0.05). The ER was measured at 96.57° before the season. It was decreased to 93.15° after the season. The decrease was statistically significant (*P*<0.001). When pitching a ball, a pitcher uses a combination of his left foot and his right hand. A successful pitch depends on dynamics, coordination, supple ROM, and muscular strength (6).

**Table 1:** A changes in DS-ROM of youth baseball pitchers

**Variable**	**Pre-season**	**Stove league**	***t***	***df***	***P***
**IR**	31.65±7.85	34.03±8.41	−2.168	25	0.04^*^
**ER**	96.57±5.57	93.15±5.99	4.237	25	0.001^***^

Values are mean ± SD.

* *P* < 0.05;

*** *P* < 0.001.

In conclusion, these results indicate that systematic collection and analysis of data on shoulder joints are needed in order to prevent youth baseball players from having injuries and improve their athletic performance. In addition, their pitching forms should be corrected to increase ball speed. Moreover, it is important to limit the number of pitches and inning so that they can get adequate rest.
